# Development and Validation of Predictors for the Survival of Patients With COVID-19 Based on Machine Learning

**DOI:** 10.3389/fmed.2021.683431

**Published:** 2021-09-22

**Authors:** Yongfeng Zhao, Qianjun Chen, Tao Liu, Ping Luo, Yi Zhou, Minghui Liu, Bei Xiong, Fuling Zhou

**Affiliations:** ^1^Department of Hematology, Zhongnan Hospital of Wuhan University, Wuhan, China; ^2^Department of Hematology, The First Affiliated Hospital of Yangtze University, Jingzhou, China; ^3^National Engineering Research Center for E-Learning, Central China Normal University, Wuhan, China; ^4^The State Key Laboratory of Biocatalysis and Enzyme Engineering of China, College of Life Sciences, Hubei University, Wuhan, China; ^5^Department of Urology, Center for Evidence-Based and Translational Medicine, Zhongnan Hospital of Wuhan University, Wuhan, China

**Keywords:** COVID-19, SARS-CoV-2, survival, machine learning, Borderline-Smote

## Abstract

**Background:** The outbreak of COVID-19 attracted the attention of the whole world. Our study aimed to explore the predictors for the survival of patients with COVID-19 by machine learning.

**Methods:** We conducted a retrospective analysis and used the idea of machine learning to train the data of COVID-19 patients in Leishenshan Hospital through the logical regression algorithm provided by scikit-learn.

**Results:** Of 2010 patients, 42 deaths were recorded until March 29, 2020. The mortality rate was 2.09%. There were 6,812 records after data features combination and data arrangement, 3,025 records with high-quality after deleting incomplete data by manual checking, and 5,738 records after data balancing finally by the method of Borderline-1 Smote. The results of 10 times of data training by logistic regression model showed that albumin, saturation of pulse oxygen at admission, alanine aminotransferase, and percentage of neutrophils were possibly associated with the survival of patients. The results of 10 times of data training including age, sex, and height beyond the laboratory measurements showed that percentage of neutrophils, saturation of pulse oxygen at admission, alanine aminotransferase, sex, and albumin were possibly associated with the survival of patients. The rates of precision, recall, and f1-score of the two training models were all higher than 0.9 and relatively stable.

**Conclusions:** We demonstrated that percentage of neutrophils, saturation of pulse oxygen at admission, alanine aminotransferase, sex, and albumin were possibly associated with the survival of patients with COVID-19.

## Introduction

Since December 2019, an ongoing outbreak of coronavirus disease 2019 (COVID-19) had struck the world, which was caused by severe acute respiratory syndrome coronavirus-2 (SARS-CoV-2) (1, 2). Coronaviruses belong to a family of single-stranded RNA viruses, which mainly cause respiratory symptoms but also some gastrointestinal symptoms, and these aggravated the severity of the disease quickly and accurately (3, 4). As for COVID-19, it is crucial to recognize the mortality risk factors of patients for timely recognition and intervention of patients who are at high risk of mortality. Several studies for exploring predictors of survival had been developed. However, most of these studies had relatively few outcome events and unbalanced samples (5, 6).

Machine learning (ML) is a kind of artificial intelligence, focusing on teaching computers to learn complex tasks and make predictions, to learn and generalize from large and complex datasets. ML algorithms include linear and logistic regression, artificial neural networks, support vector machines, tree-based methods, neural networks, and so on (7). Traditional logistic regression is the standard method for developing prediction models. However, previous comparison studies have suggested that machine learning algorithms can be more accurate than traditional logistic regression methods (8). Over the last few years, a number of advanced machine learning techniques have been developed to create predictive models (9, 10). On the other hand, the samples in Decision Trees and XGBoost were unbalanced. Borderline-1 Smote could solve the sample imbalance by an oversampling technique that synthesized a few samples.

By far there are few prognosis prediction models from the general COVID-19 population using machine learning. In the current research, we used the logical regression algorithm provided by scikit-learn to train the data of COVID-19 patients in Leishenshan Hospital.

## Methods

### Study Design and Patients

The 2010 patients with COVID-19 who were admitted to Leishenshan Hospital from February 8, 2020, to March 29, 2020, were included in our research. All patients met the diagnostic criteria of “Diagnosis and Treatment Scheme of Novel Coronavirus–Infected Pneumonia (trial 6th)” formulated by the General Office of the National Health Committee (GOoNH).

We used the logical regression algorithm provided by scikit-learn to train the clinical data of patients with COVID-19 in Leishenshan Hospital, in order to get the prediction model of survival and help clinicians change the treatment measures to improve the prognosis of patients in a timely fashion.

### Data Processing

Data processing included data preprocessing, data split, and data training. The original data was imported into Microsoft SQL Server 2014. The original table was named datalss. The data table after features conversion and features decomposion was named issfeature, which included inpatient number, feature, the value of feature, and corresponding time. The data table after features combination and data arrangement was named dataresult, which contained basic information and laboratory measurements. The original data in datalss could be matched and decomposed into multiple lines through regular expression. The inpatient number was used as the primary key to insert the decomposed results into the issfeature line by line. All the laboratory measurements in issfeature were merged with the inpatient number and corresponding time as the primary keys, excluding the data involving personal privacy. In dataresult, if the data of one feature missed more than 30%, we would delete this feature; if the data missed <30%, we would complete the data cell with certain rules. The cell could be filled in with the latest data within 3 days or the median; instead, the data over 3 days would be directly discarded. Data after preprocessing was finally split into test data-sets (25%) and training data-sets (75%).

**Figure 1:**
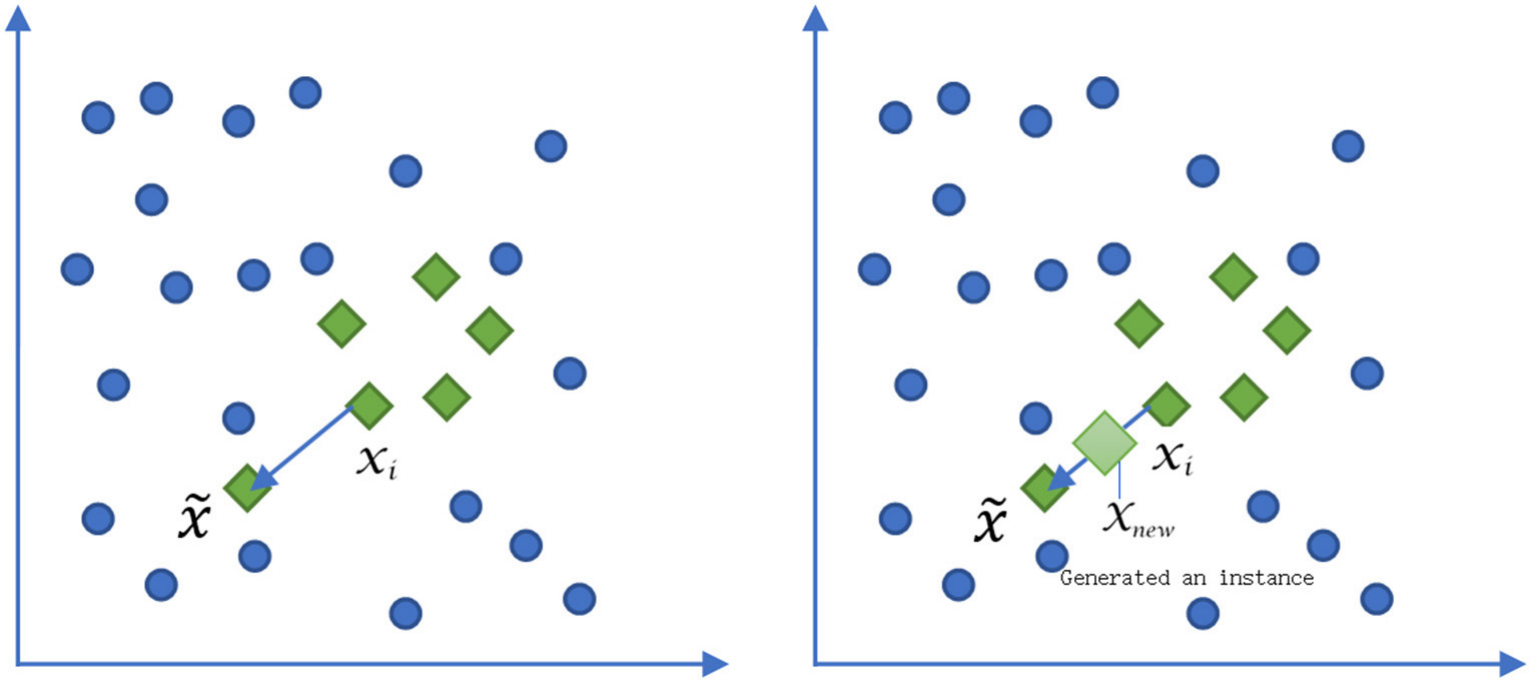
The diagram of Borderline-1 Smote algorithm to deal with the data balancing between death and survival. ***x_i_*** represented a minority sample of death. **$\tilde{x}$** was an adjacent sample of the selective minority sample. ***x_new_*** was a sample between the ***x_i_*** and $\tilde{x}$.

We used the logical regression algorithm interface provided by scikit-learn to get the prediction model of survival. Borderline-1 Smote was used to balance the data between death and survival class, the diagram of which was shown in Figure 1. Balancing data means that the data of death and survival class is roughly balanced, so as to avoid the incorrect learning of the model due to the small number of data of a certain class and the small number of “voters”.

There were four major steps for the logical regression, including setting the binary dataset space, logical regression prediction function, loss function, and solving the parameters of the prediction function. The model parameters should meet the following conditions: L2 regularization used to prevent over fitting of the model; the regularization coefficient λ = 1; tol = 1e−4, the threshold for judging the error range of iteration termination; solver = 'lbfgs', Quasi Newton method used to solve the minimum value of loss function. The evaluation indicators for the training model included precision, accuracy, recall, and f1-score. The mathematical formulas during the logical regression appear in Supplementary Table 1.

## Results

### Data Features

Of 2010 patients, 42 deaths were recorded, with a mortality rate of 2.09%. There were 93 data features in total, which included name, admission number, admission time, sex, age, height, certificate number, weight, healing or not, death or not, length of stay, stay in Intensive Care Unit, length of stay in Intensive Care Unit, length of stay after returning to normal, interleukin-1β (IL-1β), interleukin-2γ (IL-2γ), interleukin-8 (IL-8), tumor necrosis factor-a (TNF-a), interleukin-10 (IL-10), interleukin-6 (IL-6), procalcitonin (PCT), alanine aminotransferase (ALT), aspartate aminotransferase (AST), albumin, alkaline phosphatase, gamma glutamyl transpeptidase, creatine kinase, lactate dehydrogenase, total bilirubin, direct bilirubin, indirect bilirubin, total bile acid, total protein, urea nitrogen, creatinine, uric acid, total carbon dioxide, cystatin C, α-hydroxybutyrate dehydrogenase, prothrombin time (Pt), international normalized ratio, Pt-% activity, activated partial thromboplastin time, fibrinogen, thrombin time, D-dimer, leukocytes, neutrophils, percentage of neutrophils, lymphocytes, percentage of lymphocytes, monocytes, percentage of monocytes, red blood cells, hemoglobin, hematocrit, mean platelet volume, total platelet counts, serum amyloid protein A, thrombin antithrombin complex, plasmin-α 2 plasmin inhibitor complex, thrombomodulin, tissue plasminogen activator inhibitor-1 complex, severity of illness at admission, low flow oxygen inhalation at admission, high flow oxygen inhalation at admission, positive pressure oxygen supply at admission, endotracheal intubation at admission, saturation of pulse oxygen at admission, mild illness, moderate illness, serious illness, antiviral treatment, antibacterial treatment, hormone treatment, antimalarial treatment, vitamin C treatment, traditional Chinese medicine treatment, the maximum of low flow oxygen inhalation, the maximum of high flow oxygen inhalation, the maximum of positive pressure oxygen supply, the maximum of endotracheal intubation, the maximum of extracorporeal membrane oxygenation, length of extracorporeal membrane oxygenation, nutritional support, length of low flow oxygen inhalation, length of high flow oxygen inhalation, length of positive pressure oxygen supply, length of endotracheal intubation, results of nucleic acid detection, novel coronavirus antibody immunoglobulin M, novel coronavirus antibody immunoglobulin G, length of stay, and results of nucleic acid detection.

### Data Preprocessing Results

There were 207,987 records obtained in datalss. After features conversion and features decomposion, there were 13,403 records obtained in issfeature. After analysis, 6,591 records were deleted because there were nucleic acid detection records only and no other detections recorded for patients. After features combination and data arrangement, there were 6,812 records in dataresult. Finally, there were 3,025 records with high-quality after manual checking, in order to ensure valid, correct, and complete records. We used the method of Borderline-1 Smote to balance the data between death and survival samples. Finally, there were 5,738 data records obtained after data balancing. The data samples were divided into the training data-set and the test data-set in a 3 to 1 method.

### Model Training Results

The features included in the model training included glutamic pyruvic transaminase, aspartate aminotransferase, albumin, alkaline phosphatase, gamma glutamyl transpeptidase, creatine kinase, lactate dehydrogenase, total bilirubin, direct bilirubin, indirect bilirubin, total bile acid, total protein, urea nitrogen, creatinine, uric acid, total carbon dioxide, Cystatin C, α-hydroxybutyrate dehydrogenase, prothrombin time, international normalized ratio, Pt% activity, activated partial thromboplastin time, fibrinogen, thrombin time, D-dimer, leukocytes, percentage of neutrophils, lymphocytes, percentage of lymphocytes, monocytes, percentage of monocytes, red blood cells, hemoglobin, hematocrit, mean platelet volume, total platelet counts, and saturation of pulse oxygen at admission.

We carried out 10 times of model training about laboratory measurements, the scores of which were very high. The rates of precision, recall, and f1-score of the training model were all higher than 0.9 and relatively stable (Table 1). Therefore the training model was effective and data processing results were ideal. The results of model training showed that albumin, saturation of pulse oxygen at admission, alanine aminotransferase, and percentage of neutrophils were possibly associated with the survival of patients. The weight coefficients of these features were higher than 1.5 (Table 2).

**Table 1:** The scores of training model based on laboratory measurements of COVID-19 patients in Leishenshan Hospital, China.

**^#^No**	**Class**	**precision**	**recall**	**f1-score**	**support**
1	survival	0.98	0.96	0.97	716
1	Death	0.96	0.98	0.97	719
2	survival	0.99	0.96	0.97	738
2	Death	0.96	0.99	0.97	697
3	survival	0.98	0.96	0.97	734
3	Death	0.96	0.98	0.97	701
4	survival	0.98	0.95	0.97	732
4	Death	0.95	0.98	0.97	703
5	survival	0.99	0.95	0.97	724
5	Death	0.95	0.99	0.97	711
6	survival	0.98	0.96	0.97	703
6	Death	0.96	0.98	0.97	732
7	survival	0.98	0.97	0.98	720
7	Death	0.97	0.98	0.98	715
8	survival	0.99	0.95	0.97	705
8	Death	0.95	0.99	0.97	730
9	survival	0.98	0.95	0.96	748
9	Death	0.95	0.98	0.96	687
10	survival	0.97	0.96	0.96	718
10	Death	0.96	0.97	0.96	717
	average value	0.97	0.97	0.97	

# *The numbers of “1 to 10” represented the number of times of training models*.

**Table 2:** The results of model training based on laboratory measurements of COVID-19 patients in Leishenshan Hospital, China.

**No**	**Data Features**	**^#^Absolute value of weight coefficient**
1	Albumin	2.178
2	SpO2 at admission	1.853
3	Alanine aminotransferase	1.717
4	Percentage of neutrophils	1.650
5	Creatinine	1.291
6	Mean platelet volume	1.207
7	Direct bilirubin	1.141
8	Cystatin C	1.103
9	Indirect bilirubin	1.096
10	Percentage of lymphocytes	0.999
11	Lactate dehydrogenase	0.981
12	Total bilirubin	0.804
13	Red blood cells	0.791
14	Aspartate aminotransferase	0.760
15	Absolute value of lymphocyte	0.690
16	Total protein	0.690
17	Uric acid	0.593
18	Pt % activity	0.570
19	Hemoglobin	0.541
20	Alkaline phosphatase	0.347
21	Hematocrit	0.335
22	Total carbon dioxide	0.334
23	Percentage of monocytes	0.332
24	Gamma glutamyl transpeptidase	0.312
25	Activated partial thromboplastin time	0.308
26	Urea nitrogen	0.284
27	Creatine kinase	0.264
28	Prothrombin time	0.263
29	White blood cells	0.231
30	D-dimer	0.200
31	Absolute value of neutrophils	0.200
32	Absolute value of monocytes	0.152
33	Fibrinogen	0.146
34	Total bile acids	0.113
35	International normalized ratio	0.105
36	Total platelet counts	0.094
37	Thrombin time	0.027
38	α-hydroxybutyrate dehydrogenase	0.015

# *The absolute value of weight coefficient represented contribution of the features to the model prediction or the embodiment of the importance*.

**Figure 2:**
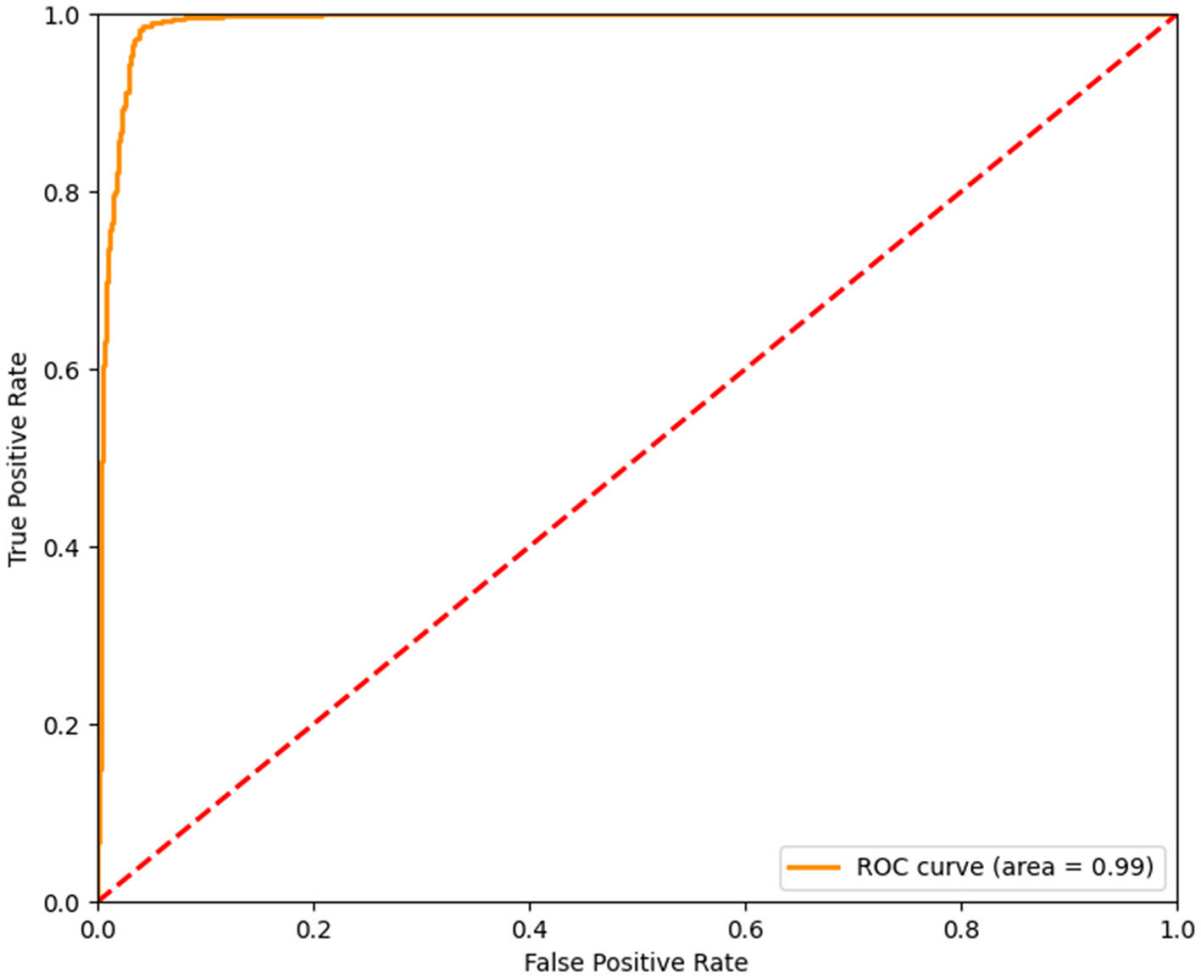
The AUC of Model Training. The AUC of the model training based on the logical regression algorithm provided by scikit-learn was higher than 0.9. The abscissa represented the false positive rate, the ordinate represented the true positive rate. The AUC of the training model was 0.99. The closer the AUC was to 1, the better the accuracy of training model was.

In order to avoid bias and obtain a relatively stable accuracy in the results, we carried out another 10 times of model training about the features including age, gender, and height beyond the laboratory measurements, the scores of which were very high. The rates of precision, recall, and f1-score were all higher than 0.9 (Table 3). Moreover, the area under curve (AUC) was higher than 0.9 (Figure 2). Therefore the training model was effective and data processing results were ideal. The results of model training showed that percentage of neutrophils, saturation of pulse oxygen at admission, alanine aminotransferase, sex, and albumin were possibly associated with the survival of patients. The weight coefficients of these features were higher than 1.5 (Table 4).

**Table 3:** The scores of model training based on the features of COVID-19 patients including age, sex, and height in Leishenshan Hospital, China.

**^#^No**	**class**	**precision**	**recall**	**f1-score**	**support**
1	survival	0.99	0.96	0.97	724
1	death	0.96	0.99	0.97	711
2	survival	0.99	0.97	0.98	744
2	death	0.97	0.99	0.98	691
3	survival	0.98	0.97	0.98	732
3	death	0.97	0.98	0.97	703
4	survival	0.99	0.97	0.98	752
4	death	0.96	0.99	0.98	683
5	survival	0.98	0.97	0.97	714
5	death	0.97	0.98	0.97	721
6	survival	0.99	0.97	0.98	731
6	death	0.97	0.99	0.98	704
7	survival	1	0.96	0.98	719
7	death	0.96	1	0.98	716
8	survival	0.98	0.97	0.98	714
8	death	0.97	0.98	0.98	721
9	survival	0.99	0.96	0.97	718
9	death	0.96	0.99	0.98	717
10	survival	0.97	0.97	0.97	748
10	death	0.97	0.97	0.97	687
	average value	0.98	0.98	0.98	

# *The numbers of “1 to 10” represented the number of times of training models*.

**Table 4:** The results of model training based on the features of COVID-19 patients including age, sex, and height in Leishenshan Hospital, China.

**No**	**Data Features**	**^#^Absolute value of weight coefficient**
1	Percentage of neutrophils	1.936
2	SpO2 at admission	1.904
3	Alanine aminotransferase	1.825
4	Sex	1.574
5	Albumin	1.474
6	Percentage of lymphocytes	1.3721
7	Indirect bilirubin	1.351
8	Lactate dehydrogenase	1.334
9	Direct bilirubin	1.286
10	Weight	1.282
11	Creatinine	1.096
12	Cystatin C	1.010
13	Mean platelet volume	0.983
14	Total bilirubin	0.813
15	Activated partial thromboplastin time	0.808
16	Creatine kinase	0.808
17	Age	0.787
18	Pt -% activity	0.736
19	Percentage of monocytes	0.705
20	Absolute value of lymphocyte	0.676
21	Total protein	0.566
22	Red blood cell	0.511
23	Uric acid	0.507
24	Hemoglobin	0.506
25	Absolute value of monocytes	0.395
26	Thrombin time	0.256
27	Absolute value of neutrophils	0.217
28	Alkaline phosphatase	0.2143
29	Hematocrit	0.168
30	Total bile acids	0.159
31	Aspartate aminotransferase	0.097
32	Gamma glutamyl transpeptidase	0.079
33	Fibrinogen	0.067
34	Prothrombin time	0.065
35	Total carbon dioxide	0.060
36	D-dimer	0.056
37	Total platelet count	0.054
38	α-hydroxybutyrate dehydrogenase	0.035
39	Urea nitrogen	0.024
40	White blood cells	0.018
41	International normalized ratio	0.009

# *The absolute value of weight coefficient represented contribution of the features to the model prediction or the embodiment of the importance*.

## Discussion

Prediction of disease outcome is one of the most interesting and challenging tasks for physicians. Multiple logistic regression was traditionally used to analyze the factors associated with an outcome in a variety of disciplines (11). In general, for linear characteristic variables, logistic regression is a very efficient algorithm, because the variables are independent of each other. Instead, for nonlinear characteristic variables, there will be interactions between them, and logistic regression is not an ideal algorithm. On the other hand, for developing prediction factors, many studies have proved that logistic regression provided by machine learning is superior to traditional logistic regression (8). Machine learning has become a powerful tool for medical researchers. This technique can discover and identify the associations from complex and large datasets. Decision Tree is one of decision-making methods which uses the tree of probability and graph theory to compare different schemes in decision-making (12). The machine learning methods of Random Forest and XGBoost were used to rank clinical features for mortality risk (6). However, the samples in the above models including Decision Trees and XGBoost were unbalanced. Borderline-1 Smote could solve the sample imbalance problem by oversampling technique that synthesized a few samples.

We applied the logical regression algorithm provided by scikit-learn to obtain the influencing factors related to the survival of patients with COVID-19. Borderline-1 Smote was used to solve the data imbalance between death and survival patients. The rates of precision, recall, and f1-score of the training model were very high. The results of 10 data training showed that percentage of neutrophils, saturation of pulse oxygen at admission, alanine aminotransferase, sex, and albumin were possibly associated with the survival of COVID-19 patients.

One survival analysis revealed that male was associated with death in patients with severe COVID-19, together with older age, leukocytosis, high lactate dehydrogenase level, cardiac injury, hyperglycemia, and high-dose corticosteroid use (13). There was one review that summarized the latest clinical and epidemiological evidences for gender and sex differences in COVID-19 patients (14). The results in our study were consistent with these results. ACE2 was identified as a receptor for the spike protein of SARS-CoV that facilitated viral entry into target cells and was abundantly expressed in airway epithelial cells and vascular endothelial cells (15, 16). Therefore, some researchers speculated that ACE2 was possibly related to the severity of patients with COVID-19, and even a hypothesis of using inhibitors that block both ACE and ACE2 zinc metalloproteases and their downstream pathways in these patients was proposed (17). One study suggested that Angiotensin-converting enzyme 2 (ACE2) expression of the kidney was higher in males than females due to the presence of testosterone and estrogen regulatory activities on post-translational mechanisms (18). However, whether the relevance of sex with the survival of patients with COVID-19 was through ACE2 remains to be further proved, and further histological and pathology studies are needed to examine the influence of sex on the expression of lung ACE-2 and the survival of patients with COVID-19.

A retrospective cohort study was conducted in 140 patients with moderate to severe COVID-19, and the results showed that hypoxemia was associated with in-hospital mortality (19). The levels of saturation of pulse oxygen at admission could predict the prognosis of severe COVID-19 patients (20). Comparing to non-severe cases, severe cases tended to have lower level of serum albumin and saturation of pulse oxygen. Hypoalbuminemia was associated with the outcomes of COVID-19 patients (21). It was also confirmed in our study that saturation of pulse oxygen at admission and albumin were associated with the survival of COVID-19 patients. In our study, the percentage of neutrophils was also associated with the survival of COVID-19 patients. The results of 32 hospitalized patients who were critically ill with confirmed COVID-19 compared with 67 noncritically ill patients showed that lower neutrophils and lymphocytes could be used for early detection and identification of critically ill patients (22). A systematic review proved stronger correlations of neutrophils (OR = 17.56) with COVID-19 mortality than with SARS or MERS mortality (23). These results were consistent with the results in our study based on artificial intelligence. Zhang JJY et al. carried out one meta-analysis that showed ICU admission was predicted by increased alanine aminotransferase, aspartate transaminase, and elevated lactate dehydrogenase (24). A high AST/ALT ratio on admission was an independent risk factor for poor prognosis of COVID-19 patients (25). AST abnormality was associated with the highest mortality risk compared with the other indicators of liver injury during hospitalization (26). The association of ALT with the survival of COVID-19 patients was also proved in our study, not other indicators of liver injury.

The main limitation of our study is that the sample size is not big enough. If the sample size is large enough, then the results of the data training model will be closer to the real situation. In the future, we will make it into a web application, publish it on the internet for others to predict, and further improve the model.

In conclusion, the results of our study which used machine learning demonstrated that percentage of neutrophils, saturation of pulse oxygen at admission, alanine aminotransferase, sex, and albumin were possibly associated with the survival of patients with COVID-19, with very high accuracy of the prediction model and balance between data. These results need to be focused on and could help clinicians to identify the risk factors related to death in time and make timely treatment for patients.

## Data Availability Statement

The original contributions presented in the study are included in the article/Supplementary Files, further inquiries can be directed to the corresponding author/s.

## Ethics Statement

Written informed consent was not obtained from the individual(s) for the publication of any potentially identifiable images or data included in this article.

## Author Contributions

YZ wrote the manuscript. QC, TL, PL, YZ, and ML collected the data. YZ, QC, and TL analyzed the data. BX and FZ designed the project, provided professional guidance, and revised the manuscript. All authors contributed to the article and approved the submitted version.

## Funding

This work was supported by the National Key Research and Development Program of China (No. 2020YFC0845500).

## Conflict of Interest

The authors declare that the research was conducted in the absence of any commercial or financial relationships that could be construed as a potential conflict of interest.

## Publisher's Note

All claims expressed in this article are solely those of the authors and do not necessarily represent those of their affiliated organizations, or those of the publisher, the editors and the reviewers. Any product that may be evaluated in this article, or claim that may be made by its manufacturer, is not guaranteed or endorsed by the publisher.
