# Supplementary material for: Development and Validation of Predictors for the Survival of Patients With COVID-19 Based on Machine Learning
Source: Front Med (Lausanne). 2021 Sep 22;8:683431. doi: 10.3389/fmed.2021.683431 (PMC8493244; doi:10.3389/fmed.2021.683431)
Supplement: Supplementary file 1 [file Table_1.DOCX]

| **TABLE 1 The Mathematical Formulas for the Logical Regression by SMOTE Algorithm** | |
| --- | --- |
| **Steps** | **Specific Formulas** |
| **Setting the binary dataset space** | $\mathbf{D=}\left( \mathbf{x}_{\mathbf{1}}\mathbf{,}\mathbf{y}_{\mathbf{1}} \right)\mathbf{,}\left( \mathbf{x}_{\mathbf{1}}\mathbf{,}\mathbf{y}_{\mathbf{1}} \right)\mathbf{,}\left( \mathbf{x}_{\mathbf{1}}\mathbf{,}\mathbf{y}_{\mathbf{1}} \right)\boldsymbol{,\ldots}\left( \mathbf{x}_{\mathbf{1}}\mathbf{,}\mathbf{y}_{\mathbf{1}} \right)\mathbf{,}\mathbf{x}_{\mathbf{1}}\boldsymbol{\subseteq}\mathbf{R}^{\mathbf{n}}\mathbf{,}\mathbf{y}_{\boldsymbol{i\in}}\boldsymbol{0,1,i=1,2,3\ldots N}$ |
| **Prediction function** | $\boldsymbol{h}_{\boldsymbol{\theta}}\left( \boldsymbol{x} \right)\boldsymbol{=}\frac{\boldsymbol{1}}{\boldsymbol{1+}\boldsymbol{e}^{\boldsymbol{-(\theta}^{\boldsymbol{T}}\boldsymbol{x+1)}}}$ |
| **Loss function** | $\mathbf{cost}\left( \mathbf{h}_{\boldsymbol{\theta}}\left( \mathbf{x} \right)\mathbf{,y} \right)\mathbf{=-y*}\log\left( \mathbf{h}_{\boldsymbol{\theta}}\left( \mathbf{x} \right) \right)\mathbf{-}\left( \mathbf{1-y} \right)\mathbf{*log(1-}\mathbf{h}_{\boldsymbol{\theta}}\left( \mathbf{x} \right)\mathbf{)}$ |
| **Solving the parameters of prediction function** | $\boldsymbol{\theta}^{\boldsymbol{(k+1)}}\boldsymbol{=}\boldsymbol{\theta}^{\boldsymbol{(k)}}\boldsymbol{-}{\boldsymbol{\nabla}^{\boldsymbol{2}}\boldsymbol{J}\left( \boldsymbol{\theta}^{\boldsymbol{(k)}} \right)}^{\boldsymbol{-1}}\boldsymbol{\nabla J}\left( \boldsymbol{\theta}^{\boldsymbol{(k)}} \right)$ |

**Supplementary Table1**. **The Mathematical Formulas for the Logical Regression by**

**SMOTE Algorithm**
